# Heated tobacco products: another tobacco industry global strategy to slow progress in tobacco control

**DOI:** 10.1136/tobaccocontrol-2018-054340

**Published:** 2018-09-12

**Authors:** Stella A Bialous, Stanton A Glantz

**Affiliations:** 1 Center for Tobacco Control, UCSF, San Francisco, California, USA; 2 Social and Behavioral Sciences, School of Nursing UCSF, San Francisco, California, USA; 3 Division of Cardiology, University of California, San Francisco, San Francisco, California, USA

**Keywords:** tobacco industry, global health, non-cigarette tobacco products, public policy

## Abstract

There has been a global decline in tobacco consumption that, if continued, will negatively impact the tobacco industry’s profits. This decline led the industry to invent and market new products, including heated tobacco products (HTP). HTP are an extension of the industry’s strategies to undermine government’s tobacco regulatory efforts as they are being promoted as part of the solution for the tobacco epidemic. Under the moniker of ‘harm reduction’, the tobacco companies are attempting to rehabilitate their reputation so they can more effectively influence governments to roll back existing tobacco control policies or create exemptions for their HTP. Rolling back tobacco control policies will make it easier for the companies to renormalise tobacco use to increase social acceptability for all their products. When regulations are absent or when loopholes exist in classifying HTP as a tobacco product (thus subject to all tobacco control regulations), the industry’s marketing of HTP is making these products more visible to the public and more accessible. Governments need to ensure that HTP are regulated as tobacco products or drugs and reject partnerships with the tobacco companies to promote ‘harm reduction’. The tobacco companies remain the vector of the tobacco-caused epidemic and cannot be part of the global tobacco control solution.

## Introduction

As of April 2018, Philip Morris International (PMI), British American Tobacco (BAT) and Japan Tobacco International (JTI) were aggressively promoting their ‘heated tobacco products’ (HTP, also called ‘heat-not-burn’ heated tobacco, smoke-free tobacco and other ‘less risky’ products around the world (table 1). Marketing for these products and media accounts of HTP launches in different countries explicitly state or imply that they are safer than cigarettes.^1–6^ In a few instances, marketing materials claim that HTP are potentially helpful to smokers who want to quit.^6^

**Table 1:** Availability of HTP by major cigarette company and country of availability (January 2018)

Company	Product	Year launched	Countries/Comments
British American Tobacco^91^	iFuse* glo	2015 2016	Romania, Japan, Switzerland, Canada, South Korea, Russia
China National Tobacco Corporation / SMTA^92^	Not reported	Not launched	A few of the companies claim to have over 30 patents of HTP and continue to be engaged in research and development of these products. But none yet are in the market.
Imperial Brands^93^	Not reported	Not launched	Focusing on e-cigarettes at the moment, claims to have options to launch when it deems that time is right
Japan Tobacco International^31^	Ploom TECH†	2016	Japan, Switzerland
KT&G Corp.^94^	lil	2017	South Korea
Philip Morris International‡ 6	IQOS TEEPS§	2014 Not yet launched	Canada, Guatemala, Colombia, Czech Republic, Denmark, France, Germany, Greece, Israel, Italy, Kazakhstan, Lithuania, Monaco, Netherlands, Poland, Portugal, Romania, Russia, Serbia, Slovak Republic, Slovenia, Spain, Switzerland, Ukraine, UK, South Africa, South Korea, Japan, New Zealand

*It is unclear that iFuse will remain in the market in Romania, where Glo was introduced in 2018.

†Ploom TECH is described as a hybrid between a HTP and a vaporiser. It is to be used with Mevius capsules. Mevius is one of JTI’s best-selling cigarette brands. The capsules contain tobacco which are then heated by vapour.

‡PMI website states that it is developing a new heated nicotine delivery product that has no tobacco, STEEM, among other ‘reduced risk’ products.

§ We do not know what TEEPS stands for, it is not included in the product’s description (https://www.pmi.com/smoke-free-products/teeps-carbon-heated-tobacco-product).

HTP, heated tobacco product.

In the USA, claims of reduced risk (what US law calls a Modified Risk Tobacco Product or MRTP) must be approved by the Food and Drug Administration (FDA) before a tobacco company can market a product with reduced exposure or risk claims.^7^ In December 2016, PMI submitted a request to the FDA to market IQOS, one of its HTP as a MRTP, claiming that it is a reduced risk tobacco product. The application to sell in the USA without these claims falls under a different process.^7^ In particular, despite evidence to the contrary in their MRTP application,^8–10^ PMI claimed that smokers who switch completely to IQOS would experience a reduction in the health-related risks associated with smoking.^11 12^ In January 2018, the FDA Tobacco Product Scientific Advisory Committee recommended against FDA approval of reduced risk claims for IQOS.^13^ This paper provides an overview of the global HTP market, the marketing claims that tobacco companies are making when promoting HTP, and the policy implications of HTP within the context of the tobacco industry’s ongoing efforts to disrupt tobacco control progress. IQOS and the other HTP products represent a continuation of the tobacco industry’s documented strategies to undermine effective tobacco control, including successful implementation of the WHO Framework Convention on Tobacco Control (FCTC).

### Plans to rapidly introduce heated tobacco products

As of April 2018, the industry was rapidly introducing new HTP.^14 15^ In December 2014, PMI became the first company to make a large-scale launch of HTP, promoting IQOS. In Italy, rapid market penetration led to an increase in IQOS use, including intent to use IQOS among non-smokers and long-term former smokers who would otherwise remain tobacco-free.^16 17^ In the case of never smokers, HTP has the potential to cause harm, despite the tobacco companies’ claim to the contrary.^16^ The finding that non-smokers and former smokers are using IQOS illustrates how the introduction of HTP can compound the harms caused by other tobacco products.

PMI built a US$120 million production facility in Switzerland and announced, in June 2017 the building of a US$320 million facility in Germany^5^ focused entirely on the development and production of HTP. PMI announced plans to double production capacity from 50 billion heatsticks (the disposable tobacco stick that fits in the IQOS device) in 2017 to 100 billion sticks in 2018.^15^ In Japan, IQOS quickly gained market share, reaching 10% of the tobacco market in less than 1 year. In 2017, JTI responded with the launch of Ploom TECH,^1^ followed by BAT’s glo.^18^

We do not know the exact number of countries where the tobacco industry is seeking approval to introduce HTPs in 2018, but a 115 page 2014 presentation by PMI Research and Development titled ‘Reduced Risk Products Briefing’^19^ released by Reuters^14^ indicates that PMI aimed to reach 50 markets by the end of 2018. It appears that PMI selected the top 50 markets after considering volume of cigarettes sold, existing product regulation, the ‘economical, political and legal environment’ and likelihood of commercial success.^19^ BAT stated on its website in 2017 that it planned to have its ‘potentially reduced risk products’ in 40 markets by the end of 2018.^4^ In April 2018, PMI shares dropped in value subsequent to its announcement of an earlier than expected plateauing of the Japanese IQOS market.^20^

### Regulatory considerations

A 2017 Reuters investigation found that before launching IQOS in a country, PMI engaged with high level government officials in attempts to convince regulators that IQOS had health benefits and therefore should not be subject to the same regulatory restrictions as cigarettes, including marketing, labelling and taxation.^14^ As Martin King, PMI’s Asia President told *Asia Times* in a 2017 interview: ‘Ensuring the right market infrastructure and regulatory frameworks are in place is essential to our overall launch schedule for Asia. Fundamentally, any potentially less-harmful alternative to cigarettes needs to be recognised by regulators and consumers as different from cigarettes—taxed differently, labelled differently, and with the freedom to communicate the product attributes openly; only then can smokers have the information they need to encourage them to switch to a smoke-free alternative’.^2^ Similarly, in 2017 Ruth Dempsey, PMI’s Director for Regulatory and Scientific Affairs, told the Costa Rican newspaper *Imprensa Libre* that existing regulations in some countries make it difficult for PMI to launch IQOS and suggested that countries needed to change their regulatory frameworks to allow PMI to communicate with consumers and explain the advantages of IQOS.^3^

In 2017 in Colombia, the Vice President of PMI affiliate Coltobaco, Humberto Mora, lamented that legislation they supposed to treat HTP differently than other tobacco products did not pass a Senate Committee. He stated that lacking specific regulation, the company’s goal was to ensure that minors did not buy the product.^21^ Mora also claimed that HTP did not generate any toxic components associated with cardiovascular diseases and cancer.^22^

In March 2017, the Ministry of Health of Israel allowed IQOS to enter the market without any restrictions that are applicable to cigarettes and exempted from the tax scheme for other tobacco products.^23^ These decisions generated a strong protest from health advocacy groups who filed a court case to protest the Ministry’s decision.^24^ The announcement also ran counter previous statements by the Health Ministry’s legal advisor. In January 2018, the Ministry reversed its position and convinced the Minister of Finance to announce that HTPs would be taxed similarly to cigarettes.^25^

As of April 2018, there were a range of regulatory approaches to HTP and most of the countries being targeted by the industry for launching HTP were facing the challenge of regulating HTP under existing tobacco control laws that may not explicitly include HTP, which may have made it easier for the companies to open up loopholes in existing laws to evade regulations that apply to all other tobacco products. At a minimum, all claims of harm reduction must be proven with robust, independent evidence,^26^ and all regulatory measures of the FCTC should be applicable to the packaging, taxation, sales and marketing of HTP.^7^

### Marketing heated tobacco products

Marketing of these products, and claims being made about them, need to be regulated.^7 27^ In 2016 in Japan, the appearance of IQOS in a popular television programme was followed by a rapid increase in IQOS use, highlighting the need to regulate HTP marketing and use.^28^ The agency that represented the TV celebrities that included IQOS on their television show stated that ‘they received absolutely no payment from Philip Morris or affiliated companies’ to discuss IQOS on their show’.^29^ In Canada, where marketing restrictions exists, PMI is using a series of direct to consumers marketing strategies, including events, and claims of a ‘smoke-free future’, highlighting the need for governments to develop regulatory framework around marketing claims.^30^

The tobacco companies are using a series of claims in the marketing of HTP. Both in websites and statements to the media and investors, HTP are presented as less harmful but not risk-free. Some media accounts of product launches state that HTP reduce the levels of harmful tobacco components by 90%–95% compared with cigarettes, while others emphasise the lack of odour or visible emissions as part of marketing campaigns. It is important to note that as of April 2018, there is no evidence to confirm this claimed 90%–95% lower level of harm. Other marketing claims highlight that these products produce no smoke, that is, are smoke-free. Implied in these claims, in ads and stores globally, is that smokers should switch from cigarettes to these new, allegedly less harmful, products.

#### Reduced harm

In a July 2017 press release, JTI also claimed a 99% reduction on a list of tobacco product constituents that have been identified as harmful by WHO’s Tobacco Product Regulation Expert Group.^31^ In a December 2017 press release, BAT made a similar claim for its HTP, glo, in Romania, where in addition to the 90%–95% reduction in harmful components, BAT claimed that the new product was aligned with WHO’s recommendations for regulating tobacco products content.^32^ BAT qualified the 90%–95% claim with a footnote stating that this was based on an analysis of nine ‘harmful components’ in cigarettes that the WHO had identified as target for reduction. WHO responded with a statement in February 2018, stating that WHO was ‘in no way endorsing BAT’s product nor the company’s claims concerning the product’.^33^

#### Smoke-free

In 2017 in South Africa,^34 35^ PMI emphasised HTP as ‘smoke-free’ in its marketing. At the opening of an IQOS store in Cape Town PMI capitalising on the fact that South African law does not require 100% smoke-free public places (by allowing for designated smoking areas), Blaine Dodds, Head of Marketing for Reduced Risk Products at Philip Morris South Africa stated that the company was

extremely excited to partner with these malls which have agreed to allow the trial of this product indoors. The HeatSticks or heated tobacco units inserted in the IQOS device are not ignited, only heated and therefore do not generate smoke. The indoor air quality is not negatively impacted by the aerosol. This affords PMSA the opportunity to leverage the area of the store to demonstrate a smoke-free future to South Africans.^34^

A footnote in the press release that quotes Dodds states that ‘IQOS is not risk free. The best way to reduce tobacco related health risks is to quit tobacco use altogether’.^34^

A June 2017 JTI press release emphasised the lack of odour from Ploom TECH in an effort to ensure that indoor use is not restricted.^1^

In sum, by 2018, the tobacco companies were promoting HTP, globally as a reduced harm product and an option to address the tobacco epidemic. As in previous attempts of the tobacco industry to be a stakeholder in tobacco control,^36–39^ these marketing efforts were providing the tobacco companies with access to decision makers and opinion leaders, continuing the industry’s efforts to influence the policy process to protect its profits.

### Scientific and political engagement

The tobacco companies use HTP products as part of their broader political and public relations activities to position them as ‘partners’ to address the tobacco epidemic rather than as the vectors that are causing it. This is a similar strategy previously used by the tobacco industry to promote itself as a partner of public health in reducing the harms of tobacco, while obfuscating the scientific evidence pointing that harm reduction is achieved through tobacco control policies that decrease consumption.^39^

PMI’s 2014 internal ‘10 year Corporate Affairs Objectives and Strategies’^40^ released as part of a series of investigations by Reuters outlines PMI’s strategies to support its ‘combustible and reduced risk (RPP) product businesses’. The strategy document provided a series of examples of activities to renormalise its business to regain access to the political and policy discussions related to tobacco control. One of the key objectives was to ‘establish PMI as a trusted and indispensable partner, leading its sector and bringing solutions to the table’. Another key objective was to ‘define and pave the way for the right fiscal and regulatory frameworks to secure PMI’s RPP portfolio as the pathway for future growth’.^40^

According to this ‘confidential internal use only’ plan, PMI’s ‘external engagement’ plans were:

1. Establish the concept of harm reduction as legitimate public policy in tobacco regulation.
2. Establish the legitimacy of tobacco companies to be a part of the regulatory debate on RRPs [reduced risk products](‘part of solution’).
3. Leverage PMI’s innovation and scientific research to establish credibility with stakeholders.
4. Identify and engage non-traditional third party stakeholders/allies (e-cigarette manufacturers and retailers, adult consumers of RPP products, tobacco harm reduction advocates, scientific community) globally and locally.
5. Develop compelling messages and materials to support our advocacy on RPP issues.
6. Amplify and leverage the debate on harm reduction around global events (eg, COP6).
7. Continue to engage with regulators globally.^40^

As discussed below, these strategies echo the tobacco industry’s decades-long efforts to undermine tobacco control and present itself as an ‘indispensable’ partner in all policy discussions.

**Figure 1:**
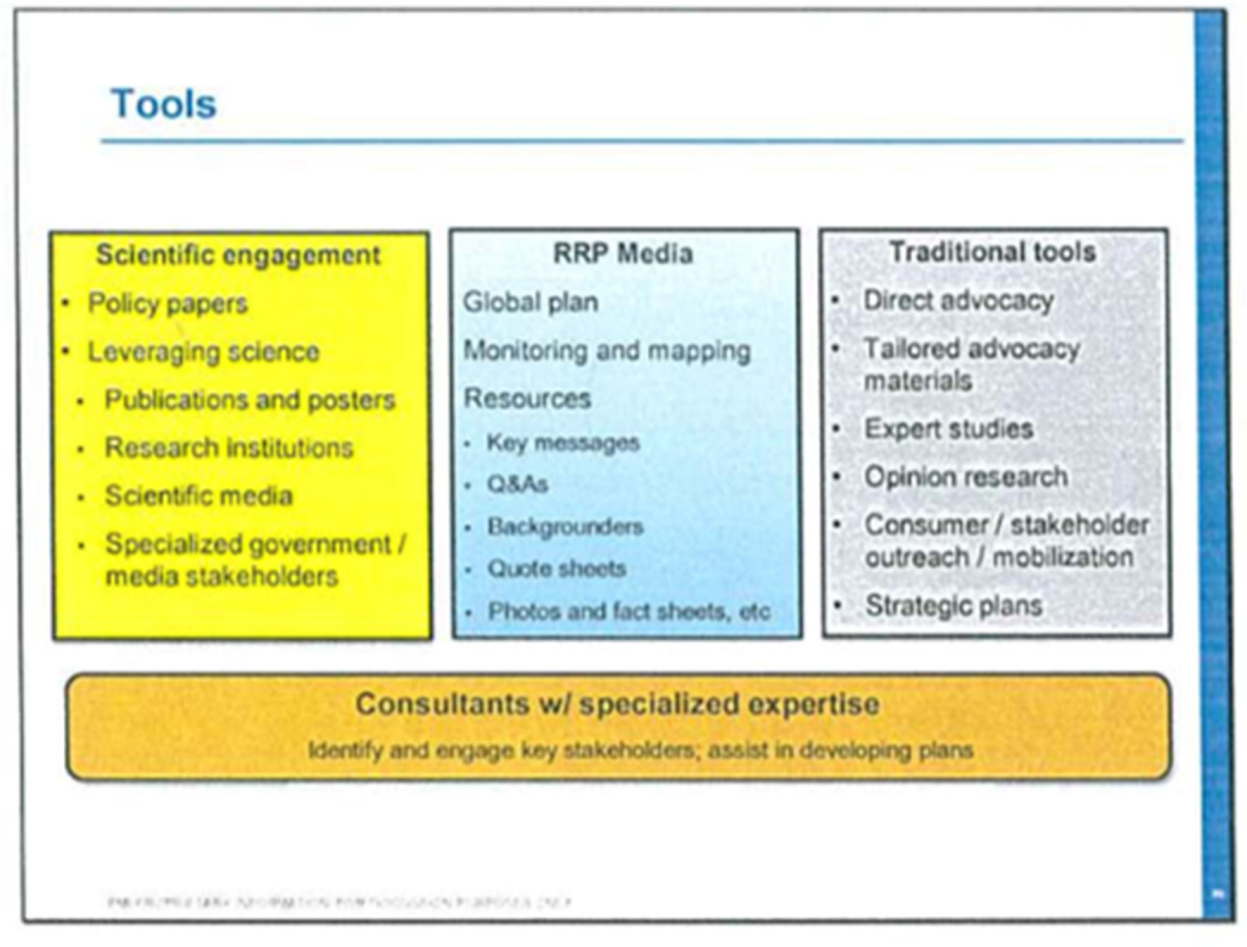
PMI’s tools to expand access to markets for its alleged reduced risk products (Slide 22 of a 125 slide presentation titled ‘Reduced Risk Products Briefing’^18^) released by Reuters^14^ as part of a series of reports on PMI activities. PMI, Philip Morris International.

The 2014 presentation by PMI titled ‘Reduced Risk Products Briefing’^19^ released by Reuters^14^ (figure 1) described how PMI planned to invoke the tobacco industry’s usual tools to influence the scientific and policy debate around tobacco control: funding of science, global media and public relations campaigns, use of consultants and support for individuals and groups that it perceives as adequate spokespeople for the company’s message.^41–46^

PMI released a full-page advertisement in newspapers on 2 January 2018 in the UK claiming that PMI was ‘trying to give up cigarettes’.^47^ In the ad, PMI explicitly expressed a desire to partner with local and national governmental authorities to support cessation services, including seeking ‘governmental; approval to insert, directly into our cigarette packs, information on quitting and on switching’.^47^ The advertisement did not mention HTP directly, but did pledge to ‘expand the availability of new, alternative products in the UK’.^47^ PMI also launched a website, nominally to communicate with smokers about quitting regular cigarettes called ‘smoke-free future’ (only available for consumers in the UK as of April 2018). PMI’s communications surrounding HTP emphasised the company’s nominal goal of a smoke free future, which is similar to the name of the Foundation for a Smoke-Free World PMI created and funded in 2017.

### Foundation for a smoke-free world

As an apparent element of PMI’s plan to expand the market for its HTP as well as rehabilitate the company’s reputation, in 2017 PMI committed almost US$1 billion (US$80 million per year for 12 years^48^) to create the Foundation for a Smoke-Free World.^49^ The foundation website stated that its goal was to ultimately eliminate smoking worldwide and ‘advancing the dialogue on smoking cessation and harm reduction’.^49^ The foundation website also stated that it was in the process of developing a research agenda, after which it would release a call for research proposals. The new foundation has a strong goal of promoting HTP as a harm reduction alternative to smoking, in alignment with PMI’s strategy to engage with the scientific community and ‘amplify’ the debate on harm reduction.^36 37^

PMI’s motives for creating the foundation were questioned by every major health authority group in the world, including the WHO, Union for International Cancer Control and the Union.^50–52^ In January 2018, the deans of 17 schools of public health in the USA and Canada issued a statement declaring that their school would not collaborate with the foundation because they considered funding from the Foundation as being funding from the tobacco industry, which these schools have rejected. Several scholars^37 53^ identified the foundation as another tobacco industry public relations campaign, similar to previous foundations or research institutes the industry had created in the past to serve its political and public relations needs. The criticism also focused on the questionable independence of the foundation from PMI^54^ and questioned the real intent behind the foundation’s research agenda. Like its predecessor organisations, the foundation captured a few scientists and academics to promote an agenda that overlaps significantly with PMI’s agenda, although research awards had not been announced as of April 2018.

## Discussion

The launching of the latest incarnation of HTPs is a reprise^55^ of similar efforts in the past to use similar products to undermine tobacco control, particularly efforts that present the tobacco industry as a harm reduction partner.

As early as the 1960s, the tobacco companies developed alternative tobacco products with the goal of supplementing the cigarette market with products. A few of these products, such as RJ Reynolds (now Reynolds America, part of BAT) *Premier* and *Eclipse* and Philip Morris’ *Accord* and *HeatBar* were marketed but received poor ratings from customers, were commercial failures and were withdrawn.^55^ It is possible the companies were not more aggressive in making ‘reduced harm’ claims on new products because of legal concerns: Claiming that the new products were safer would amount to an admission that cigarettes were dangerous, opening the door for litigation and political difficulties for the tobacco industry, including FDA regulation of new products and cigarettes in the USA.^56–58^ In addition, the FCTC did not ban cigarettes, one of the tobacco industry’s fears. All these factors laid the foundation for the wave of HTP reduced risk claims in several countries that accompanied new HTP products starting around 2014. The introduction of these new products may also have been a response to the growing popularity of e-cigarettes beginning around 2007 after independent companies introduced them before the major multinational tobacco companies entered the e-cigarettes market.^55 57^ Furthermore, the global decline of cigarette consumption and decrease in adult smoking prevalence (from 24% in 2007 to 21% in 2015), combined with the success of tobacco control, including implementation of the FCTC,^59–62^ may also have lead the tobacco companies to consider alternative products to protect their profits and political interests. HTP serves both purposes by keeping consumers using the companies’ tobacco products while providing the industry with an avenue to lobby for exemptions from FCTC and similar national regulations by claiming that HTP would be good for public health.^58^ The PMI announcement in the UK^47^ has been identified as integral to the overall tobacco industry strategy to present a changed image to the public while continuing to promote nicotine addiction.^36^

In the 1990s, with growing pressure from litigation in the USA and increasing engagement of the WHO in supporting tobacco control globally, the tobacco industry worked to create divisions within tobacco control while seeking to reposition itself politically as part of the ‘solution’ to the problems created by tobacco use.^44^ Philip Morris’ Project Sunrise, initiated in 1995, outlined a clear strategy to target certain individuals within the tobacco control community, question their credibility and integrity and work with them to promote alternative policy options that would be less harmful to the interests of the tobacco industry.^38^ Project Sunrise implemented Philip Morris’ 10-year strategy to position itself as a ‘responsible’ company and a partner in tobacco control efforts, which would give heightened access to decision makers and the possibility to influence tobacco control regulations. Despite Philip Morris’ efforts, global tobacco control did advance, with the FCTC entering into force in 2005.^43^

Since Project Sunrise, the tobacco industry has deployed a range of strategies to interfere with tobacco control, as described by the WHO.^45 46^ Among these strategies are efforts to create an image of ‘social responsibility’ and a commitment to work in partnership with governments to advance tobacco control, although neither of these initiatives have had any impact other than a public relations campaign for the tobacco industry.^46 63–66^ Another significant strategy the tobacco industry used in the early 2000s was to promote voluntary, self-regulation in an effort to prevent the FCTC from entering into force. This voluntary self-regulation focused on marketing and youth smoking prevention programmes (YSP). Both voluntary marketing regulation and industry-sponsored YSP have been demonstrated to be ineffective in addressing the tobacco epidemic.^43 67–71^

An integral part of the tobacco industry’s efforts is to promote a variety of its products in ways that imply, overtly or not, that they pose less harm than conventional cigarettes. Such misleading discourse accompanied the launch of cigarette filters, machine-measured lower-tar cigarettes, non-cigarette tobacco products such as snus and other smokeless tobacco.^39 72 73^ All these efforts sought to avoid marketing restrictions and influence policy makers to support self-regulation instead of a mandatory and more restrictive regulatory framework.^42 43 46^ Scientific evidence, on the other hand, demonstrated that filters, decreasing the number of cigarettes smoked a day or switching to a different type of cigarette are not viable risk reduction options. Similarly, as of 2018, the tobacco industry was producing its own science, and planning to fund scientists, in an effort to create evidence to support its claims.

However, emerging science indicated that HTP are unlikely to be any ‘healthier’ than conventional cigarettes, including scientific data submitted by PMI as part of its MRTP application to the FDA.^8 10 74–77^ The industry’s claims are often speculative, emphasising the ‘potential’ for these new products to either reduce harm or reduce risk of tobacco use.^78 79^ Additionally, research has demonstrated that despite claims that there is not burning of tobacco, pyrolysis and charring occurs when using IQOS, releasing highly toxic formaldehyde cyanohydrin.^80^ Others have shown that while there is a reduction is some toxic compounds, when comparing IQOS with regular cigarettes, these are not removed, and the clinical impact of exposure remains to be assessed.^81^ Nonetheless, the tobacco industry appears to be determined in using a ‘harm reduction’ frame in order to gain access to the policymaking table.

The tobacco industry’s use of the ‘harm reduction’ framework also serves to fracture the tobacco control movement, leaving it without a unified voice to communicate with the public, the media and with policy makers on the strategies to advance tobacco control. The concept of harm reduction has traditionally been embraced in several public health fields such as clean needles for injectable drug use and has been explored by some tobacco control experts in the past,^82^ with enthusiasm for the possibility of harm reduction growing with the widespread availability of electronic cigarettes in certain markets.^83–85^ The tobacco industry frames harm reduction as a common ground with health advocates and a possible entry point to influence legislation and regulation of tobacco products.^39 86 87^

As described by Peeters and Gilmore,^39^ the 2001 Institute of Medicine report on the potential tobacco harm reduction (that was heavily influenced by industry interests^88^) appears to have provided support for tobacco industry efforts to reframe harm reduction as a viable tobacco control policy option and, more importantly, to position itself as pivotal to achieving such harm reduction goals. Thus, in the past decade and a half, the tobacco industry became a vocal proponent of tobacco harm reduction and has invested millions of US dollars in research and development of new products, such as HTP, which the tobacco industry is now using to gain access to scientists, opinion leaders and decision makers as a ‘solution’ to address the tobacco epidemic. Elias and Ling^89^ describe the role the tobacco industry played a role in funding the earliest efforts to promote ‘clean nicotine’ for harm reduction and conclude that the tobacco industry will continue to seek endorsement from health authorities to its proposition of HTP as a ‘harm reduction’ strategy.

If HTP manufacturers were seriously concerned about addressing the tobacco epidemic, they would immediately withdraw from dozens of court cases where they are challenging governments’ right to implement policies that protect the public’s health. Moreover, none of the tobacco companies that are promoting HTP have made any effort to actually reduce tobacco harm by curtailing marketing of tobacco products and has continued to vigorously oppose tobacco control measures and the implementation of the FCTC at national, regional and international levels.

### FCTC Article 5.3

Governments that are a Party to the FCTC are urged to consider the regulatory options provided by the treaty when confronted with the tobacco industry’s pressure to enter new markets. There is nothing in the language of the treaty that precludes treating HTP as all other tobacco products (or a drug delivery system), including restriction of use in public places, applying labelling requirements, marketing restrictions and taxes.^7^ Additionally, Parties to the FCTC that choose to accept the tobacco industry as a stakeholder in addressing the tobacco epidemic are in breach of Article 5.3. Article 5.3 and its implementation guidelines^90^ clearly state that there is an ‘irreconcilable conflict of interest’ between health policy and the tobacco industry. It further states that the tobacco industry is not, and could not, be a partner of governments in the implementation of tobacco control measures. Thus, governments must not engage, or participate, in tobacco industry-led ‘harm reduction’ efforts.

## Conclusion

The introduction of the latest generation of HTP appears to be the latest chapter in the decades-old tobacco industry strategy of working to create partnerships with governments and health advocates, presenting these alleged ‘harm reduction’ products as an option to address the tobacco epidemic. While health authorities should keep an open mind if independent compelling evidence that a true harm reducing tobacco product is developed and could support a harm reduction policy strategy, they should also keep in mind that the past has demonstrated that partnerships with industry benefit the corporate interests of the tobacco industry and harms countries’ health and development. The evidence available to date does not convincingly demonstrate that the available HTPs will simply replace conventional cigarettes among current smokers without attracting youth or even that these products will substantially reduce health risks among users. Nevertheless, the tobacco industry has a well-developed media, public relations and scientific strategy to undermine tobacco control through HTP. It is reaching out to governments and scientists to co-opt them to promote HTP.^14^ LMICs, and scientists in these countries, are vulnerable to the appeal of industry funding and must be supported in resisting partnering with the industry and, for countries that are Parties to the FCTC, breaching its international commitments. It is unclear what impact, if any, multilateral trade agreements will have on the expansion of HTP markets or the regulation of these new products.

Despite the rapid introduction of HTPs, as of April 2018, the vast majority of countries did not yet have these products, which creates a window of opportunity to address the tobacco industry’s latest ‘harm reduction’ offensive. But, time is of essence. The FCTC provides a legal framework that encourages countries to take a series of measures regarding novel tobacco products, from banning entry into market, to regulating advertisement, sales, packaging and use^7^ allowing Parties to address HTP before these products enter the market in an unregulated fashion.

**What this paper adds**

- After decades of increasing, global cigarette consumption is falling following implementation of the evidence-based policies in the WHO Framework Convention on Tobacco Control (FCTC).
- The tobacco companies are promoting heated tobacco products (HTP) as harm reduction as part of their effort to be ‘part of the solution’ to the tobacco epidemic.
- The tobacco companies are using strategies that they have used for decades to fracture tobacco control and promote tobacco ‘harm reduction’ in an attempt to renormalise tobacco use.
- Tobacco companies are introducing HTP in markets with little or no regulatory or marketing restrains despite the fact that reduced risks claims are unproven and likely false.
- All FCTC regulatory measures should apply to HTP.
- Governments in countries where HTP are not available should keep them out and if allowed in the market at all should be under the strict regulatory framework defined by the FCTC.
